# Clinical and cytogenetic characteristics of patients diagnosed with Turner syndrome in a clinical genetics service: cross-sectional retrospective study

**DOI:** 10.1590/1516-3180.2020.0470.R2.110321

**Published:** 2021-06-14

**Authors:** Maurício Rouvel Nunes, Tiago Godói Pereira, Henry Victor Dutra Correia, Simone Travi Canabarro, Ana Paula Vanz, Paulo Ricardo Gazzola Zen, Rafael Fabiano Machado Rosa

**Affiliations:** I BSc. Master's Student, Postgraduate Program on Pathology, Universidade Federal de Ciências da Saúde de Porto Alegre (UFCSPA), Porto Alegre (RS), Brazil.; II Undergraduate Student, Department of Clinical Medicine, Universidade Federal de Ciências da Saúde de Porto Alegre (UFCSPA), Porto Alegre (RS), Brazil.; III Undergraduate Student, Department of Health Sciences, Pontifícia Universidade Católica do Rio Grande do Sul (PUCRS), Porto Alegre (RS), Brazil.; IV PhD. Professor, Department of Nursing, Universidade Federal de Ciências da Saúde de Porto Alegre (UFCSPA), Porto Alegre (RS), Brazil.; V PhD. Professor, Department of Nursing, Faculdades Integradas de Taquara, Taquara (RS), Brazil.; VI PhD. Professor, Departments of Clinical Medicine and Clinical Genetics, Universidade Federal de Ciências da Saúde de Porto Alegre (UFCSPA), Porto Alegre (RS), Brazil.; VII PhD. Professor, Departments of Clinical Medicine and Clinical Genetics, Universidade Federal de Ciências da Saúde de Porto Alegre (UFCSPA), Porto Alegre (RS), Brazil.

**Keywords:** Turner syndrome, Genetic diseases, inborn, Karyotype, Diagnostic techniques and procedures, X chromosome, Ullrich-Turner syndrome, Monosomy X, Genetic disorders

## Abstract

**BACKGROUND:**

Turner syndrome (TS) is a rare genetic disease. Understanding its clinical findings contributes to better management of clinical conditions.

**OBJECTIVE:**

To investigate the clinical and karyotypic characteristics of patients diagnosed with TS at two reference services for clinical genetics in southern Brazil.

**DESIGN AND SETTING:**

Retrospective cross-sectional study conducted in two clinical genetics services in Porto Alegre (RS), Brazil.

**METHODS:**

The sample consisted of 59 patients with TS diagnosed from 1993 to 2019. A review of their medical records was performed and a standard protocol was filled out.

**RESULTS:**

The average age of the patients at diagnosis was 15.9 years, and 40.7% were over 13 years old. The largest proportion of them (42.4%) had been referred from an endocrinology department and their constitution was 45,X (40.7%). The most common clinical findings were short stature (85.7%), hypoplastic/ hyperconvex nails (61.2%), low posterior hairline (52.1%) and cubitus valgus (45.8%). There was no difference regarding the presence of short stature (P = 0.5943), number of dysmorphia (P = 0.143), anatomical regions affected and malformations identified through imaging examinations (P = 1.0000), regarding the presence or absence of 45,X constitution. Only 6% of the patients had used growth hormone and 43%, estrogen.

**CONCLUSION:**

We found that, in general, patients with TS were being diagnosed late. This has important implications for their treatment. In addition, only a small proportion of the patients were undergoing further examination or evaluation, which appeared to be leading to underdiagnosis of many abnormalities.

## INTRODUCTION

Turner syndrome (TS), or Ullrich-Turner syndrome, is a genetic condition clinically characterized by findings such as short stature, hypogonadism, webbed neck, broad thorax and dysplastic nails.^1^ It has an incidence of 1/2500 female births. Among the TS-associated chromosomal constitutions, the most common is X chromosome monosomy, which is present in about 45% of the cases. Another frequent abnormality is a long-arm isochromosome of the X chromosome (10-20% of the cases), which occurs due to loss of the short arm and duplication of the X chromosome long arm. Other constitutions consist of cases of mosaicism involving a 45,X lineage associated with one or more additional cell lines, a ring X chromosome and short-arm partial deletions of the X chromosome.^2,3^

Some clinical features are associated with *SHOX* gene haploinsufficiency, such as short stature and skeletal anomalies, which can be treated with growth hormone. Patients with TS usually also present hypergonadotropic hypogonadism due to ovarian failure and require hormone replacement therapy with sex steroids.^3^

It is noteworthy that in TS there may be great variability of clinical findings. While some girls may present the classic form with the main dysmorphia that has already been described, there may be others with few findings. Thus, girls and women with the syndrome may often go unnoticed and may present late diagnosis, which has important implications for case management and treatment. Growth deficit without a known cause is considered to be one of the most important TS findings. Thus, attention can be drawn to this diagnosis through performing karyotype evaluation.^4^

## OBJECTIVE

The aim of this study was to investigate the clinical and karyotypic characteristics of patients diagnosed with TS at two reference services for clinical genetics in southern Brazil, in order to identify findings that might especially assist in making early diagnoses among these patients.

## METHODS

This was a retrospective cross-sectional study. The sample consisted of 59 patients diagnosed with TS at two clinical genetics services, one at a university and the other at a mother-and-child hospital, both in Porto Alegre (RS), Brazil, from 1993 to 2019. Patients with records presenting incomplete clinical descriptions were excluded. The study was approved by the ethics committees of the university (protocol number 2.230.086, date: August 21, 2017) and hospital (protocol number 2.326.171, date: October 10, 2017). This paper was written in accordance with the guidelines for reporting observational epidemiological studies (STROBE).

For data collection, a review of the patients' records was performed and a standard protocol was filled out. The data collected consisted of age at diagnosis, reason for referral and specialty from which the referral was made, evaluation period, GTG-banding karyotype result obtained from a peripheral blood sample, dys-morphological physical examination with anthropometric measurements and description of the dysmorphia and secondary sexual characteristics, use of growth hormone and estrogen therapy, and presence of associated diseases and abnormalities that were identified through complementary imaging examinations and expert assessments.

The patients were classified according to their age at diagnosis, as follows: 0 to < 2 years; 2 to 13 years; and > 13 years.^5^ For this, we considered the starting ages recommended by the Brazilian Ministry of Health for hormone treatments.^6^

The evaluation period was divided into two: patients attended between 1993 and the end of 2005; and those attended between the beginning of 2006 and 2019. The division into these two periods was done because public policies for supporting the diagnosis of these patients were created and medical records became computerized at the beginning of the second period.

Regarding karyotype results, the patients were divided into groups with and without the chromosomal constitution 45,X. The patients' heights and weights were evaluated using standard growth curves for females.^7^ Microcephaly was reported in relation to age (absolute microcephaly) and height (relative or true microcephaly). To evaluate the body mass index (BMI), we used the virtual calculator of the Ministry of Health's Virtual Health Library (VHL) for primary healthcare and took the patient's age into consideration (child or adult). The patients were classified according to their BMI into groups with low weight, adequate (normal) weight, overweight or obesity (https://aps.bvs.br/apps/calculadoras/?page=7 and https://aps.bvs.br/apps/calculadoras/?page=6). Dysmorphia was divided according to the anatomical region affected.^8^ Secondary sexual characteristics were described in accordance with Tanner's stages.^9^ Malformations identified through complementary imaging examinations and expert assessments were classified according to the body system involved.

Data processing and analysis were performed using the Microsoft Excel 2013 software and the Statistical Package for the Social Sciences (SPSS) software for Windows, version 2.0 (IBM Corp, Armonk, New York, United States). In the analysis, we used the two-tailed Fisher exact test and the t test for mean comparisons. P < 0.05 was considered significant.

## RESULTS

The sample consisted of 59 patients, ranging in age from 1 month to 34 years (mean of 15.9 years and median of 10.6 years). Regarding age, 16 patients (27.1%) were between 0 and < 2 years of age, 19 (32.2%) between 2 and 13 years and 24 (40.7%) > 13 years. It is noteworthy that a significant number of patients were already > 13 years old, i.e. they were beyond the age indicated for treatment with growth hormone.

Regarding referrals, the largest proportion of the patients had been referred from an endocrinology department (n = 25; 42.4%). Among the remainder, 11 (18.7%) came from pediatrics, 10 (16.9%) from gynecology, 5 (8.5%) from neonatology, 4 (6.8%) from genetics, 1 (1.7%) from gastroenterology and 1 (1.7%) from cardiology. Only a small proportion of the patients aged 0 to 13 years (n = 35) had been referred by pediatricians (25.7%). Thirty-two patients (54.2%) had already been referred with a suspicion of TS. The others had secondary amenorrhea (n = 7; 11.9%), multiple malformations (n = 4; 6.8%), primary amenorrhea (n = 3; 5.1%) and growth retardation (n = 3; 5.1%). It was noteworthy that one patient (1.7%) was referred due to suspicion of an inborn metabolism error.

The evaluation period ranged from 1993 to 2019: 45 patients (76.3%) were attended between 1993 and the end of 2005; and 14 (23.7%) between 2006 and 2019. Among the chromosomal constitutions observed, the most common was 45,X (n = 24; 40.7%). Others included 45,X/46,X,i(X)(q10) (n = 4; 6.8%); 45,X/46,X,r(X) (n = 4; 6.8%); 45,X/46,XX (n = 3; 5.1%); 46,X,del(Xq) (n = 3; 5.1%); and 46,X,del(Xp) (n = 2; 3.4%). The total number of cells analyzed ranged from 18 to 116 (average of 29.7). Structural changes to the X chromosome were observed in 27 cases (45.8%) and mosaicism in 26 (44.1%).

**Figure 1.**
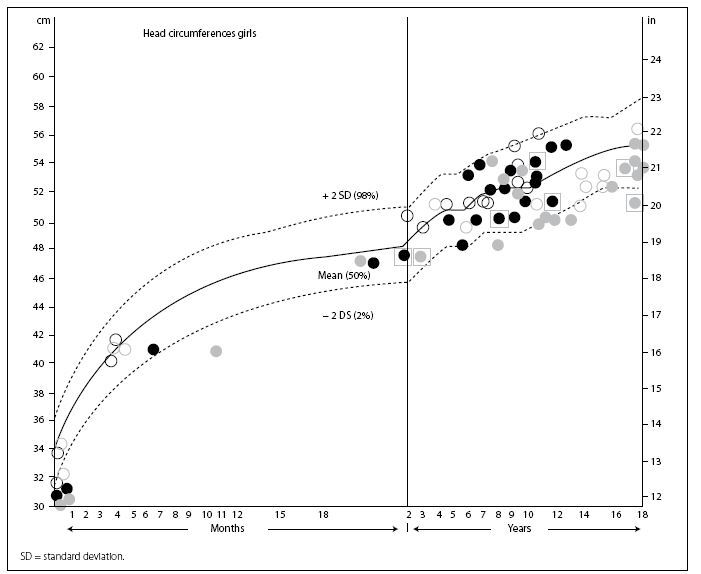
Head circumference according to age (gray circles) and height (black circles). The cases in which the circle is empty correspond to those with the constitution 45,X (the filled circles, which are the remaining cases, presented other chromosomal constitutions relating to Turner's syndrome). The cases in which the circles are inside squares are of patients who used growth hormone.

Short stature was presented by 85.7% of the patients. In 5.4% of them, short stature was the only clinical finding observed, while in 8.1% up to two types of dysmorphia were seen in association with this finding. Among the patients with absolute microcephaly (27.7%), none of them had true microcephaly (**Figure 1**). Regarding weight, the BMI showed that 18.5% were overweight and 33.3% were obese. The anatomical regions most affected were the skin, hair and nails (83.4%), limbs (70.8%) and neck (53.1%). The types of dysmorphia most frequently described in the physical examination were hypoplastic/hyperconvex nails (61.2%), low posterior hairline (52.1%) and cubitus valgus (45.8%). It was possible for the patients to present more than one phenotypic characteristic. In comparing the presence of short stature between patients with and without the constitution 45,X, we did not find any significant difference (P = 0.5943). Patients with or without constitution 45,X also did not show any significant difference regarding the number of types of dysmorphia per patient (P = 0.143). We did not find any significant association between the presence of short stature and occurrence of overweight/obesity (P = 1.0000).

In comparing the anatomical regions affected in patients with and without the constitution 45,X, we did not observe any significant association in relation to any of them. However, there appeared to be a tendency towards involvement of the eyes (P = 0.0595) and neck dysmorphia (P = 0.0753) in occurrences of the constitution 45,X. In analyzing dysmorphia alone, we also did not find any association.

Regarding treatment, out of the 33 patients with an indication for use of growth hormone, only 6% were using this, and for estrogen, only 43%. Fifteen patients out of the total sample (25.9%) were prepubescent. Among the patients aged ≥ 12 years, most had pubic hair at Tanner P1 stage (55%). The others presented P2 (15%), P3 (5%), P4 (10%) and P5 (15%). Regarding breast development, a large number of patients (70%) were in stage M1. Among the others, 6% were in M2, 12% in M3, 3% in M4 and 6% in M5.

Malformations were identified through imaging examinations in 25% of the patients. These included neural tube defects (n = 1) and cardiac (n = 9), musculoskeletal (n = 2) and urinary tract abnormalities (n = 3). It should be noted that although examinations at the clinic showed that all the patients presented gonadal abnormalities, not all of them underwent confirmatory imaging tests. We did not find any relationship between the presence of these malformations and the chromosomal constitution 45,X (P = 1.0000).

It was noteworthy that only a small proportion of the patients from the sample underwent additional examinations or evaluations, including those who made part of the follow-up protocol for patients with TS: 6.8% underwent a head computed tomography scan, 3.4% electroencephalogram, 8.5% skull radiography, 6.8% spine radiography, 33.9% hand and wrist radiography, 22% echocardiography, 30.5% abdominal ultrasound, 1.6% abdominal and pelvic computed tomography scans and 25.4% pelvic and renal ultrasound. Among additional evaluations, a cardiac assessment was performed on only 13.6% of the patients, endocrinological on 10.2%, otorhinolaryngological on 10.2%, ophthalmic on 6.8%, nephrological on 5% and gynecological on 5%.

## DISCUSSION

Rare diseases are characterized by a wide diversity of signs and symptoms and can be chronic, progressive, degenerative and even disabling, thus affecting the quality of life of families and individuals. They are defined in terms of their frequency: for example, affecting up to 1.3 people per 2,000 individuals, or 65 people per 100,000 individuals. It is noteworthy that more than 80% of them derive from genetic causes, and TS is one of these. In Brazil, there are guidelines for comprehensive care for rare-disease patients, which enables organization of access to diagnostic and therapeutic resources. Therefore, these patients with TS have a clinical protocol and therapeutic guidelines that establish the diagnostic criteria and the management and treatment algorithm.^10^

Regarding the age at diagnosis, we found in our study that the average was higher than that of a Brazilian study carried out in Sâo Paulo, in which the average was 12 years, and also higher than in studies conducted in Europe.^11,12,13^ We can highlight that 40.7% of our sample was diagnosed at more than 13 years of age, which is in agreement with another study conducted in southeastern Brazil, which found that this proportion was up to 70%.^14^

Thus, we found that the diagnosis of TS is being made late. This makes it impossible to perform important treatments, such as growth hormone treatment, which is recommended by the Brazilian Ministry of Health. Moreover, late diagnosis directly influences the evaluation and even the prognosis of the patients. In this situation, patients may not be properly investigated and the protocol and therapeutic guidelines may not be followed. Consequently, patients may present complications due to lack of investigation, resulting from abnormalities that, for example, involve internal organs. Other comorbidities may also be detected.

We therefore emphasize that it is important for pediatricians and other healthcare professionals who treat children to pay attention to the possibility that girls who have short stature of undefined cause, regardless of any presence of other associated types of dysmorphia, might have TS. Among the referrals, we noticed that only a small proportion of the patients were referred by pediatricians.^15^

Among the referred patients, more than half of them were suspected of having TS, and secondary amenorrhea was the main reason for referral. However, in other studies, short stature is described as the main reason.^16^ This finding in our study corroborates to the late referral age and diagnosis of these patients. It perhaps suggests that findings such as short stature among girls may be undervalued, since non-physiological amenorrhea is a finding present only in late adolescence and most of adulthood (excluding the time after the menopause).

In the literature, karyotype 45,X is the main chromosomal constitution described among patients with TS (40-50%),^17^ a finding similar to what was observed among our patients. Mosaicisms are found at variable frequencies, which are usually in the range of 9-56%.^18^ This is concordant with the finding from our study (44.1%). We believe that this may be related to the average number of cells analyzed, which in our case was 29.7. The American College of Medical Genetics recommends that for all individuals with suspected TS, a 20-cell karyotype should be evaluated.^19^

Structural alterations can be found in up to 31% of the patients, such as the presence of a ring X chromosome and deletion of the long arm of the X chromosome.^20^ In our study, this result was found in 45.8% of the patients, and 6% had chromosomal constitutions with mosaicism and presence of structural alterations. We highlight the presence of patients with deletion of the short and long arm of the X chromosome, in our sample. It has been established in the literature that it is mainly the patients with short-arm deletions of the X chromosome who present short stature and musculoskeletal alterations.^21^

In addition, it is important to highlight the existence of patients with TS presenting mosaicism involving a chromosomal lineage with a Y chromosome. Although we did not have patients with this chromosomal constitution in our sample, it has been described in up to 10% of patients with TS in studies with large samples. These patients have peculiarities regarding their management, since the presence of the lineage with a Y chromosome leads to a risk of gonadal malignancy. Therefore, it is very important to identify these patients, because this risk gives rise to an indication for performing prophylactic gonadectomy.^22^

Short stature was present in 85.7% of our sample, but we found that there were no differences between patients with and without chromosomal constitution 45,X, in relation to this. Furthermore, occurrence of overweight/obesity was found to be unrelated to presence or absence of this chromosomal constitution, thus corroborating the results from a study conducted in Egypt.^23^ In our study, 13.5% of the patients had up to two types of dysmorphia, including short stature, and so it is important to highlight that this is the most characteristic finding among TS patients. In our study, only 6% of the patients had made correct use of growth hormone, which was a much lower proportion than in a study conducted in Albania,^24^ in which correct use was found among 54.3% of the patients. This shows the impact that late diagnosis has on treatment for patients with TS. Growth hormone treatment for TS patients has been shown to be effective and is preferably indicated between the ages of 4-6 years, and treatment needs to be started before the ages of 12-13 years.^25^

We also did not find patients with true microcephaly. However, it is important to note that almost one third of our patients had smaller-than-expected head circumference. Thus, if age is the only factor taken into consideration (absolute microcephaly), a wrong diagnosis may often be made. Microcephaly is uncommon in TS, and only a few cases with this finding have been reported in the literature.^26^

Regarding body weight, our patients' BMI was higher than that found in a national study in Ukraine,^27^ in which 13.8% of the patients were found to be overweight and 6.9%, obese. In our study, 18.5% and 33.3% of the patients were overweight and obese, respectively. This points out the importance of having endocrinological and nutritional follow-up for these patients, since the frequency of overweight and obesity is higher in TS patients than in the general population.^28^ In addition, it is noteworthy that specific growth charts, including charts for height and for weight, already exist for individuals with TS. These growth charts can be used to monitor patients after diagnosis.^29^

Among the phenotypic manifestations, low posterior hairline, cubitus valgus and hyperconvex nails were the ones most frequently observed in our sample. These observations were in line with what had previously been described in studies in both Mexico and Brazil.^30,31^ The tendency in our study for there to be higher frequency of dysmorphia involving the eye and neck regions was noteworthy. However, we did not find any association in comparing patients with or without the chromosomal constitution 45,X, in relation to the anatomical region affected and dysmorphia. In a study by Bispo et al.^31^ in Brazil, a tendency that patients with X-chromosome monosomy would have more severe phenotypes was found. However, the genotype-phenotype correlation of the dysmorphia described, between patients with and without chromosomal constitution 45,X, remains at an initial stage, which corroborates our findings.

In our study, it was also noteworthy that among the patients aged 12 years and over, 55% were in Tanner P1 stage in relation to pubic hair and 70% in M1 in relation to breast development. This matches another finding from our sample: only 43% of the patients underwent estrogen therapy. This result is of concern, as non-treatment has other important implications, including development of osteoporosis.^32^ Thus, these patients are either not being properly managed or are not adhering to the proposed treatment.

TS is associated with several abnormalities that affect the organ systems. Cardiovascular abnormalities are reported in 50% of adult and 30% of pediatric patients. In our study, they were most frequently found through imaging examinations that the patients underwent, which was consistent with previous reports.^33,34^ However, only 22% of the patients underwent an echocardiographic evaluation, which contrasts sharply with the established recommendation that all patients with TS should undergo this evaluation at the time of diagnosis, given that cardiovascular abnormalities are considered to be one of the main causes of death among them.^35^

Complementary examinations such as hand and wrist radiography and renal ultrasound were also only infrequently done among our sample (33.9% and 25.4%, respectively). It should be noted that these tests are also recommended for patients with TS, and, for example, hand and wrist radiography is indicated by the Ministry of Health to be done within the follow-up of patients with TS, i.e. there is a recommendation that the examination should be performed periodically.

In addition, renal anomalies may be silent or asymptomatic. These may have the potential to lead to renal failure and even a need for transplantation.^36^

Moreover, we found that only 25.4% of the patients in our sample underwent pelvic ultrasound examinations. For this reason, in a large number of cases, internal genitalia and gonads could not be adequately assessed. This is important, because some patients with TS may have significant gonadal abnormalities. These may even be cancerous, as previously mentioned in relation to patients who have an associated Y chromosome lineage.^22^ Therefore, this is one more reason to emphasize the importance of carrying out complementary assessments among patients with TS.

## CONCLUSION

We conclude from the data on our sample that the largest proportion of our TS patients had a chromosomal constitution 45,X, which is in agreement with the literature, and that they are being diagnosed late, often in adulthood. As discussed, this has important implications regarding absence of growth hormone therapy and estrogen use, which can lead to severe complications, such as osteoporosis. In addition, there was poor use of screening and control examinations such as echocardiography and abdominal ultrasound among these patients. This can lead to underdiagnosing of abnormalities involving internal organs, such as congenital heart diseases or renal malformations. There may be important consequences from this, especially with regard to these patients' quality of life and even their survival. Therefore, awareness of the diagnosis and findings commonly observed in TS may provide early identification and better management of the clinical conditions associated with this syndrome.
